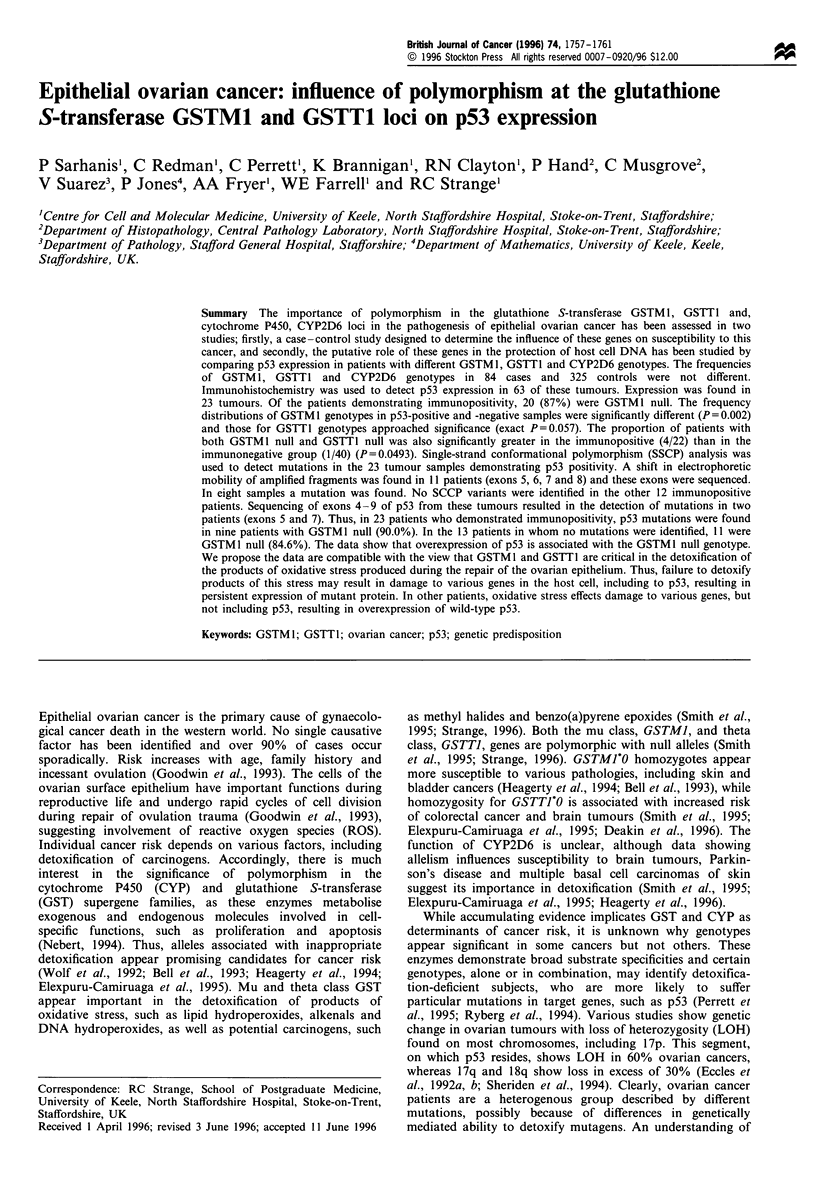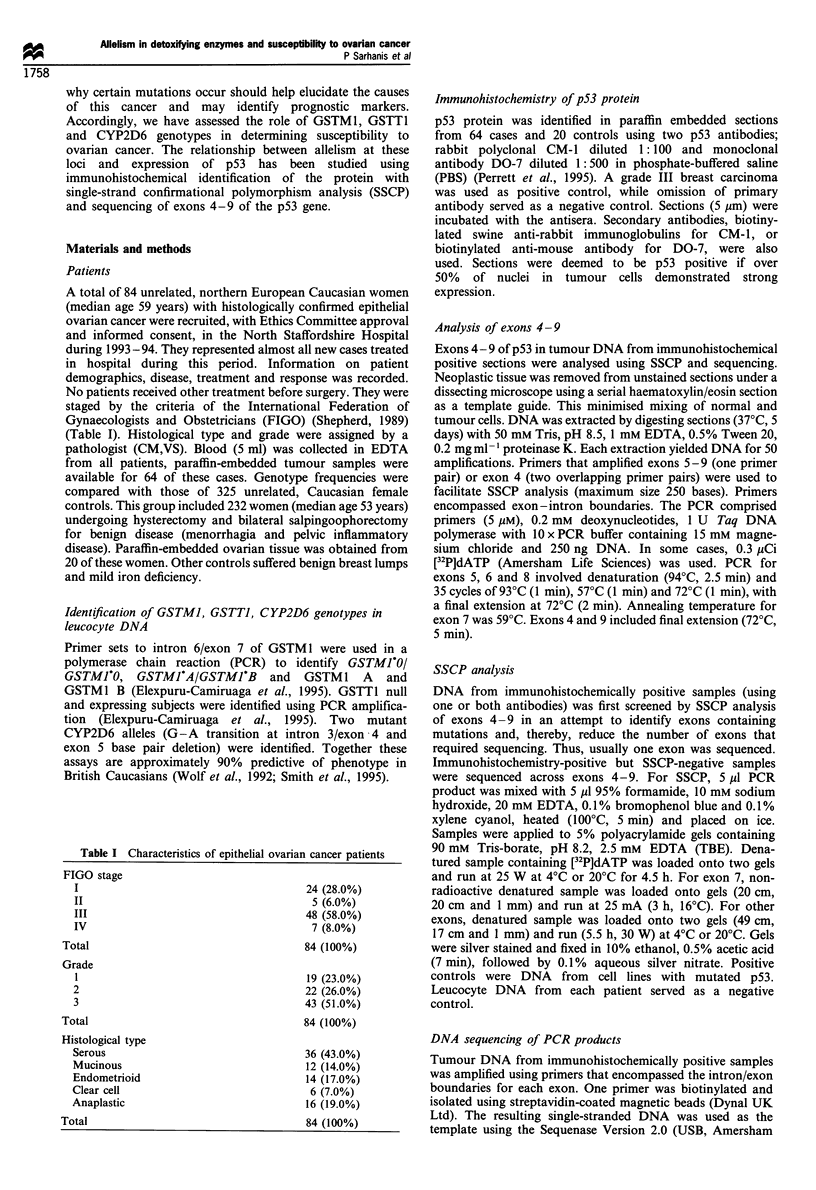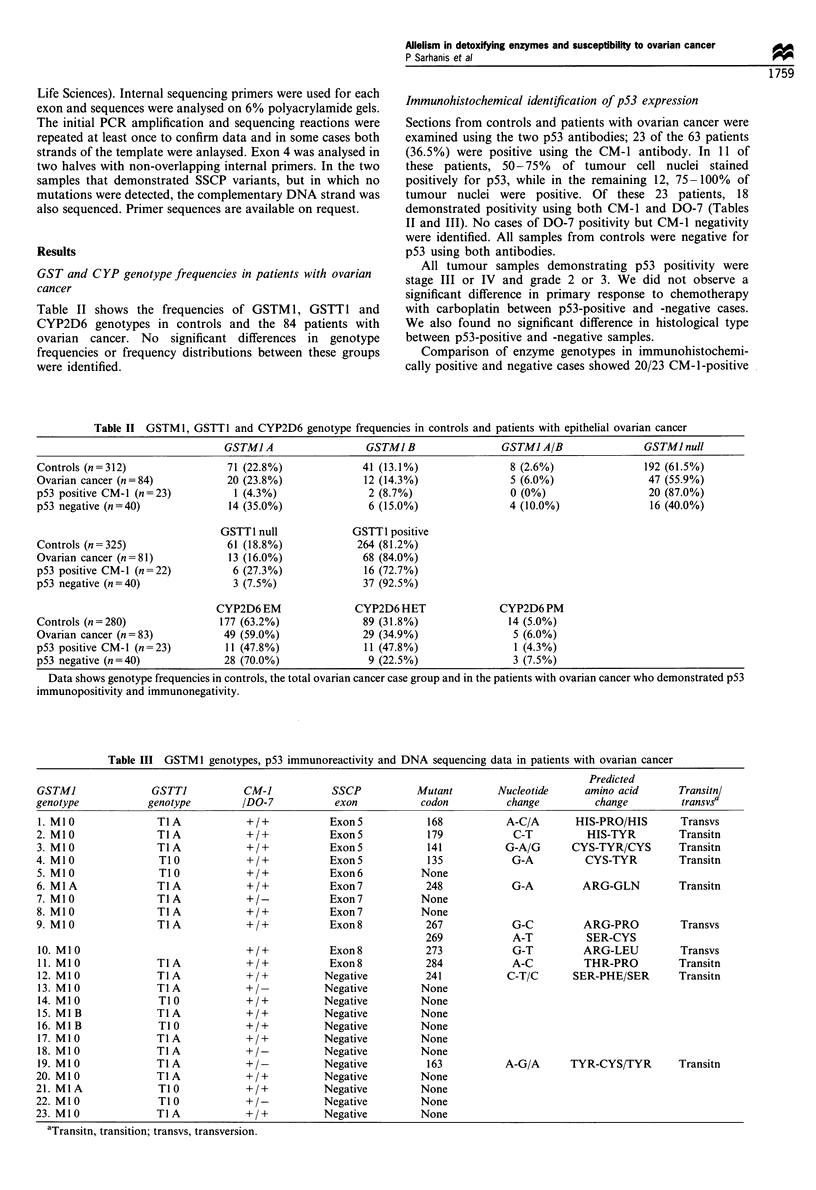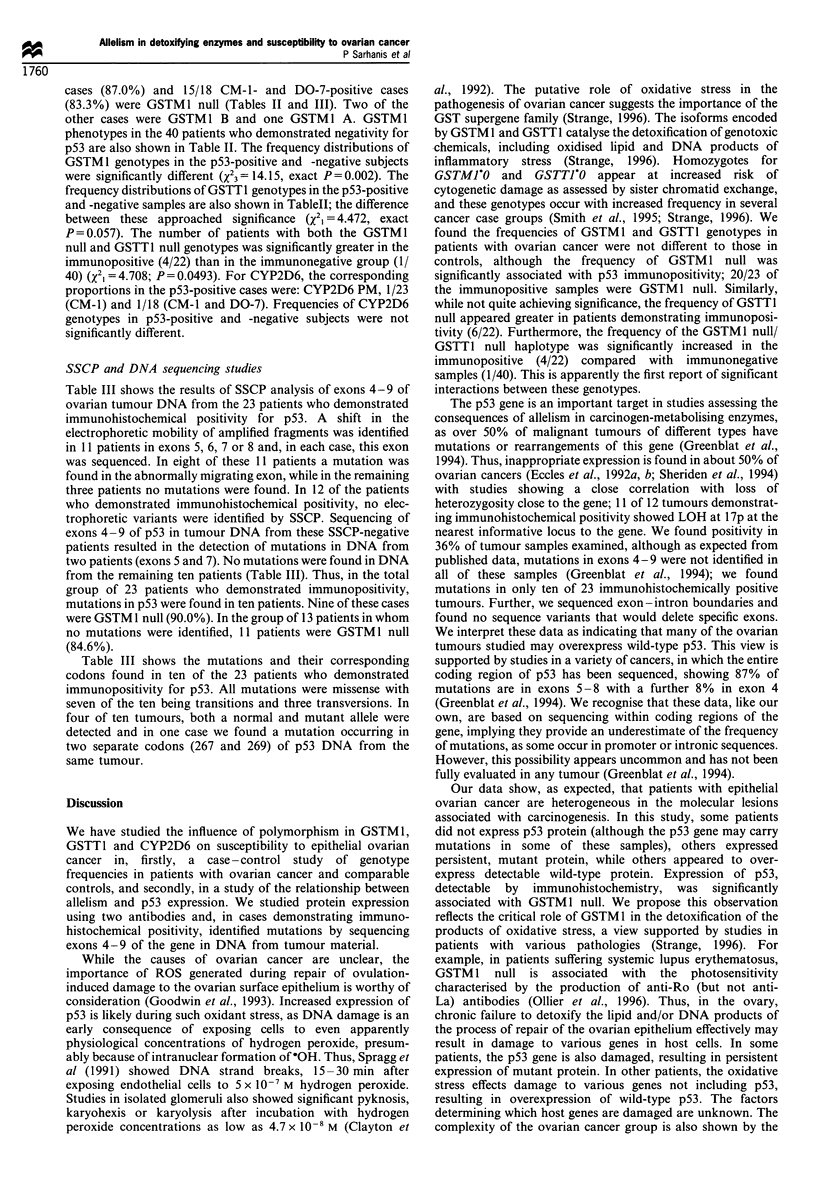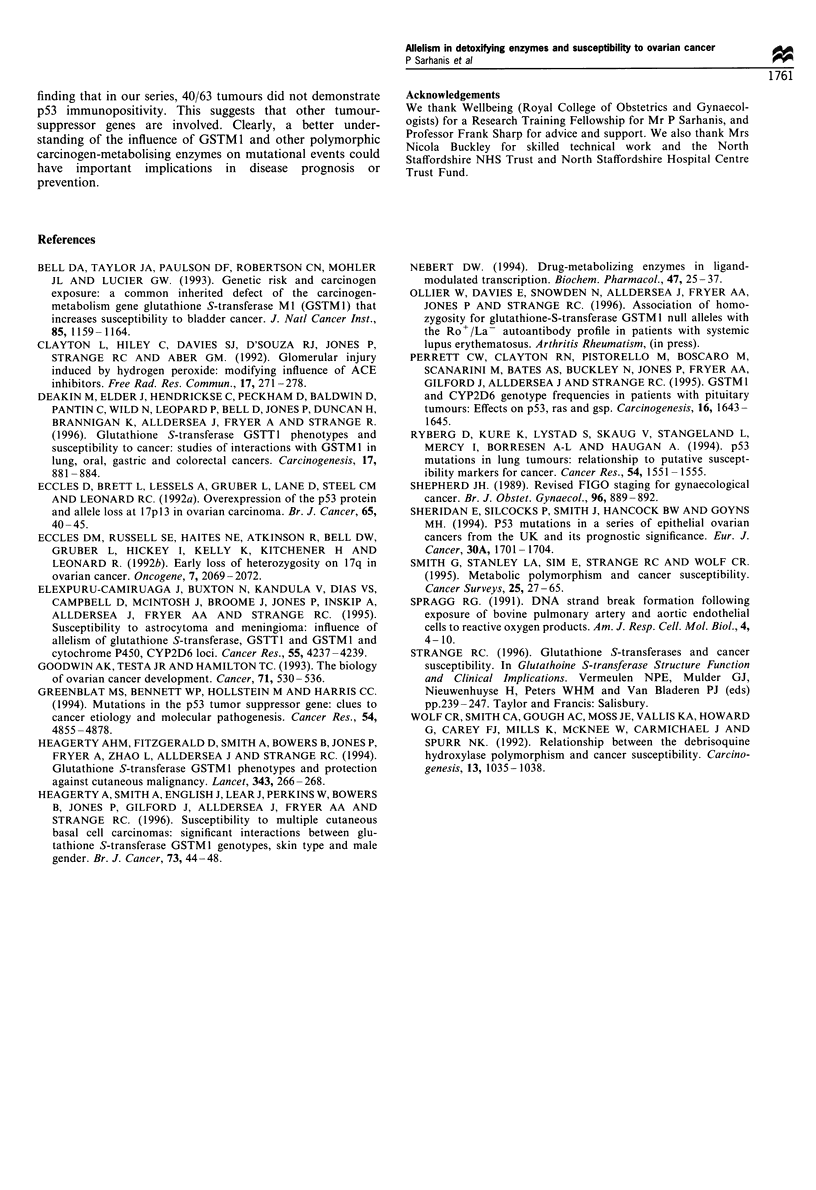# Epithelial ovarian cancer: influence of polymorphism at the glutathione S-transferase GSTM1 and GSTT1 loci on p53 expression.

**DOI:** 10.1038/bjc.1996.626

**Published:** 1996-12

**Authors:** P. Sarhanis, C. Redman, C. Perrett, K. Brannigan, R. N. Clayton, P. Hand, C. Musgrove, V. Suarez, P. Jones, A. A. Fryer, W. E. Farrell, R. C. Strange

**Affiliations:** Centre for Cell and Molecular Medicine, University of Keele, North Staffordshire Hospital, Stoke-on-Trent, UK.

## Abstract

The importance of polymorphism in the glutathione S-transferase GSTM1, GSTT1 and, cytochrome P450, CYP2D6 loci in the pathogenesis of epithelial ovarian cancer has been assessed in two studies; firstly, a case-control study designed to determine the influence of these genes on susceptibility to this cancer, and secondly, the putative role of these genes in the protection of host cell DNA has been studied by comparing p53 expression in patients with different GSTM1, GSTT1 and CYP2D6 genotypes. The frequencies of GSTM1, GSTT1 and CYP2D6 genotypes in 84 cases and 325 controls were not different. Immunohistochemistry was used to detect p53 expression in 63 of these tumours. Expression was found in 23 tumours. Of the patients demonstrating immunopositivity, 20 (87%) were GSTM1 null. The frequency distributions of GSTM1 genotypes in p53-positive and -negative samples were significantly different (P = 0.002) and those for GSTT1 genotypes approached significance (exact P = 0.057). The proportion of patients with both GSTM1 null and GSTT1 null was also significantly greater in the immunopositive (4/22) than in the immunonegative group (1/40) (P = 0.0493). Single-strand conformational polymorphism (SSCP) analysis was used to detect mutations in the 23 tumour samples demonstrating p53 positivity. A shift in electrophoretic mobility of amplified fragments was found in 11 patients (exons 5, 6, 7 and 8) and these exons were sequenced. In eight samples a mutation was found. No SCCP variants were identified in the other 12 immunopositive patients. Sequencing of exons 4-9 of p53 from these tumours resulted in the detection of mutations in two patients (exons 5 and 7). Thus, in 23 patients who demonstrated immunopositivity, p53 mutations were found in nine patients with GSTM1 null (90.0%). In the 13 patients in whom no mutations were identified, 11 were GSTM1 null (84.6%). The data show that overexpression of p53 is associated with the GSTM1 null genotype. We propose the data are compatible with the view that GSTM1 and GSTT1 are critical in the detoxification of the products of oxidative stress produced during the repair of the ovarian epithelium. Thus, failure to detoxify products of this stress may result in damage to various genes in the host cell, including to p53, resulting in persistent expression of mutant protein. In other patients, oxidative stress effects damage to various genes, but not including p53, resulting in overexpression of wild-type p53.


British Journal of Cancer (1996) 74, 1757-1761
© 1996 Stockton Press All rights reserved 0007-0920/96 $12.00

### Epithelial ovarian cancer: influence of polymorphism at the glutathione *S*-transferase GSTM1 and GSTT1 loci on p53 expression

P Sarhanis[1], C Redman[1], C Perrett[1], K Brannigan[1], RN Clayton[1], P Hand[2], C Musgrove[2], V Suarez[3], P Jones[4], AA Fryer[1], WE Farrell[1] and RC Strange[1]

[1]*Centre for Cell and Molecular Medicine, University of Keele, North Staffordshire Hospital, Stoke-on-Trent, Staffordshire;* [2]*Department of Histopathology, Central Pathology Laboratory, North Staffordshire Hospital, Stoke-on-Trent, Staffordshire;* [3]*Department of Pathology, Stafford General Hospital, Stafforshire;* [4]*Department of Mathematics, University of Keele, Keele, Staffordshire, UK.*

**Summary** The importance of polymorphism in the glutathione *S*-transferase GSTM1, GSTT1 and, cytochrome P450, CYP2D6 loci in the pathogenesis of epithelial ovarian cancer has been assessed in two studies; firstly, a case–control study designed to determine the influence of these genes on susceptibility to this cancer, and secondly, the putative role of these genes in the protection of host cell DNA has been studied by comparing p53 expression in patients with different GSTM1, GSTT1 and CYP2D6 genotypes. The frequencies of GSTM1, GSTT1 and CYP2D6 genotypes in 84 cases and 325 controls were not different. Immunohistochemistry was used to detect p53 expression in 63 of these tumours. Expression was found in 23 tumours. Of the patients demonstrating immunopositivity, 20 (87%) were GSTM1 null. The frequency distributions of GSTM1 genotypes in p53-positive and -negative samples were significantly different ($P=0.002$) and those for GSTT1 genotypes approached significance (exact $P=0.057$). The proportion of patients with both GSTM1 null and GSTT1 null was also significantly greater in the immunopositive (4/22) than in the immunonegative group (1/40) ($P=0.0493$). Single-strand conformational polymorphism (SSCP) analysis was used to detect mutations in the 23 tumour samples demonstrating p53 positivity. A shift in electrophoretic mobility of amplified fragments was found in 11 patients (exons 5, 6, 7 and 8) and these exons were sequenced. In eight samples a mutation was found. No SCCP variants were identified in the other 12 immunopositive patients. Sequencing of exons 4–9 of p53 from these tumours resulted in the detection of mutations in two patients (exons 5 and 7). Thus, in 23 patients who demonstrated immunopositivity, p53 mutations were found in nine patients with GSTM1 null (90.0%). In the 13 patients in whom no mutations were identified, 11 were GSTM1 null (84.6%). The data show that overexpression of p53 is associated with the GSTM1 null genotype. We propose the data are compatible with the view that GSTM1 and GSTT1 are critical in the detoxification of the products of oxidative stress produced during the repair of the ovarian epithelium. Thus, failure to detoxify products of this stress may result in damage to various genes in the host cell, including to p53, resulting in persistent expression of mutant protein. In other patients, oxidative stress effects damage to various genes, but not including p53, resulting in overexpression of wild-type p53.

**Keywords:** GSTM1; GSTT1; ovarian cancer; p53; genetic predisposition

Epithelial ovarian cancer is the primary cause of gynaecological cancer death in the western world. No single causative factor has been identified and over 90% of cases occur sporadically. Risk increases with age, family history and incessant ovulation (Goodwin *et al.*, 1993). The cells of the ovarian surface epithelium have important functions during reproductive life and undergo rapid cycles of cell division during repair of ovulation trauma (Goodwin *et al.*, 1993), suggesting involvement of reactive oxygen species (ROS). Individual cancer risk depends on various factors, including detoxification of carcinogens. Accordingly, there is much interest in the significance of polymorphism in the cytochrome P450 (CYP) and glutathione *S*-transferase (GST) supergene families, as these enzymes metabolise exogenous and endogenous molecules involved in cell-specific functions, such as proliferation and apoptosis (Nebert, 1994). Thus, alleles associated with inappropriate detoxification appear promising candidates for cancer risk (Wolf *et al.*, 1992; Bell *et al.*, 1993; Heagerty *et al.*, 1994; Elexpuru-Camiruaga *et al.*, 1995). Mu and theta class GST appear important in the detoxification of products of oxidative stress, such as lipid hydroperoxides, alkenals and DNA hydroperoxides, as well as potential carcinogens, such as methyl halides and benzo(a)pyrene epoxides (Smith *et al.*, 1995; Strange, 1996). Both the mu class, *GSTM1*, and theta class, *GSTT1*, genes are polymorphic with null alleles (Smith *et al.*, 1995; Strange, 1996). *GSTM1*0* homozygotes appear more susceptible to various pathologies, including skin and bladder cancers (Heagerty *et al.*, 1994; Bell *et al.*, 1993), while homozygosity for *GSTT1*0* is associated with increased risk of colorectal cancer and brain tumours (Smith *et al.*, 1995; Elexpuru-Camiruaga *et al.*, 1995; Deakin *et al.*, 1996). The function of CYP2D6 is unclear, although data showing allelism influences susceptibility to brain tumours, Parkinson's disease and multiple basal cell carcinomas of skin suggest its importance in detoxification (Smith *et al.*, 1995; Elexpuru-Camiruaga *et al.*, 1995; Heagerty *et al.*, 1996).

While accumulating evidence implicates GST and CYP as determinants of cancer risk, it is unknown why genotypes appear significant in some cancers but not others. These enzymes demonstrate broad substrate specificities and certain genotypes, alone or in combination, may identify detoxification-deficient subjects, who are more likely to suffer particular mutations in target genes, such as p53 (Perrett *et al.*, 1995; Ryberg *et al.*, 1994). Various studies show genetic change in ovarian tumours with loss of heterozygosity (LOH) found on most chromosomes, including 17p. This segment, on which p53 resides, shows LOH in 60% ovarian cancers, whereas 17q and 18q show loss in excess of 30% (Eccles *et al.*, 1992*a*, *b*; Sheriden *et al.*, 1994). Clearly, ovarian cancer patients are a heterogenous group described by different mutations, possibly because of differences in genetically mediated ability to detoxify mutagens. An understanding of

Correspondence: RC Strange, School of Postgraduate Medicine, University of Keele, North Staffordshire Hospital, Stoke-on-Trent, Staffordshire, UK
Received 1 April 1996; revised 3 June 1996; accepted 11 June 1996

why certain mutations occur should help elucidate the causes of this cancer and may identify prognostic markers. Accordingly, we have assessed the role of GSTM1, GSTT1 and CYP2D6 genotypes in determining susceptibility to ovarian cancer. The relationship between allelism at these loci and expression of p53 has been studied using immunohistochemical identification of the protein with single-strand confirmational polymorphism analysis (SSCP) and sequencing of exons 4–9 of the p53 gene.

### Materials and methods

### *Patients*

A total of 84 unrelated, northern European Caucasian women (median age 59 years) with histologically confirmed epithelial ovarian cancer were recruited, with Ethics Committee approval and informed consent, in the North Staffordshire Hospital during 1993–94. They represented almost all new cases treated in hospital during this period. Information on patient demographics, disease, treatment and response was recorded. No patients received other treatment before surgery. They were staged by the criteria of the International Federation of Gynaecologists and Obstetricians (FIGO) (Shepherd, 1989) (Table I). Histological type and grade were assigned by a pathologist (CM,VS). Blood (5 ml) was collected in EDTA from all patients, paraffin-embedded tumour samples were available for 64 of these cases. Genotype frequencies were compared with those of 325 unrelated, Caucasian female controls. This group included 232 women (median age 53 years) undergoing hysterectomy and bilateral salpingoophorectomy for benign disease (menorrhagia and pelvic inflammatory disease). Paraffin-embedded ovarian tissue was obtained from 20 of these women. Other controls suffered benign breast lumps and mild iron deficiency.

### *Identification of GSTM1, GSTT1, CYP2D6 genotypes in leucocyte DNA*

Primer sets to intron 6/exon 7 of GSTM1 were used in a polymerase chain reaction (PCR) to identify *GSTM1*0/GSTM1*0*, *GSTM1*A/GSTM1*B* and GSTM1 A and GSTM1 B (Elexpuru-Camiruaga *et al.*, 1995). GSTT1 null and expressing subjects were identified using PCR amplification (Elexpuru-Camiruaga *et al.*, 1995). Two mutant CYP2D6 alleles (G–A transition at intron 3/exon 4 and exon 5 base pair deletion) were identified. Together these assays are approximately 90% predictive of phenotype in British Caucasians (Wolf *et al.*, 1992; Smith *et al.*, 1995).

**Table I** Characteristics of epithelial ovarian cancer patients

| | |
|---|---|
| FIGO stage | |
| I | 24 (28.0%) |
| II | 5 (6.0%) |
| III | 48 (58.0%) |
| IV | 7 (8.0%) |
| Total | 84 (100%) |
| Grade | |
| 1 | 19 (23.0%) |
| 2 | 22 (26.0%) |
| 3 | 43 (51.0%) |
| Total | 84 (100%) |
| Histological type | |
| Serous | 36 (43.0%) |
| Mucinous | 12 (14.0%) |
| Endometrioid | 14 (17.0%) |
| Clear cell | 6 (7.0%) |
| Anaplastic | 16 (19.0%) |
| Total | 84 (100%) |

### *Immunohistochemistry of p53 protein*

p53 protein was identified in paraffin embedded sections from 64 cases and 20 controls using two p53 antibodies; rabbit polyclonal CM-1 diluted 1:100 and monoclonal antibody DO-7 diluted 1:500 in phosphate-buffered saline (PBS) (Perrett *et al.*, 1995). A grade III breast carcinoma was used as positive control, while omission of primary antibody served as a negative control. Sections (5 μm) were incubated with the antisera. Secondary antibodies, biotinylated swine anti-rabbit immunoglobulins for CM-1, or biotinylated anti-mouse antibody for DO-7, were also used. Sections were deemed to be p53 positive if over 50% of nuclei in tumour cells demonstrated strong expression.

### *Analysis of exons 4–9*

Exons 4–9 of p53 in tumour DNA from immunohistochemical positive sections were analysed using SSCP and sequencing. Neoplastic tissue was removed from unstained sections under a dissecting microscope using a serial haematoxylin/eosin section as a template guide. This minimised mixing of normal and tumour cells. DNA was extracted by digesting sections (37°C, 5 days) with 50 mM Tris, pH 8.5, 1 mM EDTA, 0.5% Tween 20, 0.2 mg $ml^{-1}$ proteinase K. Each extraction yielded DNA for 50 amplifications. Primers that amplified exons 5–9 (one primer pair) or exon 4 (two overlapping primer pairs) were used to facilitate SSCP analysis (maximum size 250 bases). Primers encompassed exon–intron boundaries. The PCR comprised primers (5 μM), 0.2 mM deoxynucleotides, 1 U *Taq* DNA polymerase with 10 × PCR buffer containing 15 mM magnesium chloride and 250 ng DNA. In some cases, 0.3 μCi [$^{32}$P]dATP (Amersham Life Sciences) was used. PCR for exons 5, 6 and 8 involved denaturation (94°C, 2.5 min) and 35 cycles of 93°C (1 min), 57°C (1 min) and 72°C (1 min), with a final extension at 72°C (2 min). Annealing temperature for exon 7 was 59°C. Exons 4 and 9 included final extension (72°C, 5 min).

### *SSCP analysis*

DNA from immunohistochemically positive samples (using one or both antibodies) was first screened by SSCP analysis of exons 4–9 in an attempt to identify exons containing mutations and, thereby, reduce the number of exons that required sequencing. Thus, usually one exon was sequenced. Immunohistochemistry-positive but SSCP-negative samples were sequenced across exons 4–9. For SSCP, 5 μl PCR product was mixed with 5 μl 95% formamide, 10 mM sodium hydroxide, 20 mM EDTA, 0.1% bromophenol blue and 0.1% xylene cyanol, heated (100°C, 5 min) and placed on ice. Samples were applied to 5% polyacrylamide gels containing 90 mM Tris-borate, pH 8.2, 2.5 mM EDTA (TBE). Denatured sample containing [$^{32}$P]dATP was loaded onto two gels and run at 25 W at 4°C or 20°C for 4.5 h. For exon 7, non-radioactive denatured sample was loaded onto gels (20 cm, 20 cm and 1 mm) and run at 25 mA (3 h, 16°C). For other exons, denatured sample was loaded onto two gels (49 cm, 17 cm and 1 mm) and run (5.5 h, 30 W) at 4°C or 20°C. Gels were silver stained and fixed in 10% ethanol, 0.5% acetic acid (7 min), followed by 0.1% aqueous silver nitrate. Positive controls were DNA from cell lines with mutated p53. Leucocyte DNA from each patient served as a negative control.

### *DNA sequencing of PCR products*

Tumour DNA from immunohistochemically positive samples was amplified using primers that encompassed the intron/exon boundaries for each exon. One primer was biotinylated and isolated using streptavidin-coated magnetic beads (Dynal UK Ltd). The resulting single-stranded DNA was used as the template using the Sequenase Version 2.0 (USB, Amersham

Life Sciences). Internal sequencing primers were used for each exon and sequences were analysed on 6% polyacrylamide gels. The initial PCR amplification and sequencing reactions were repeated at least once to confirm data and in some cases both strands of the template were anlaysed. Exon 4 was analysed in two halves with non-overlapping internal primers. In the two samples that demonstrated SSCP variants, but in which no mutations were detected, the complementary DNA strand was also sequenced. Primer sequences are available on request.

### Results

### *GST and CYP genotype frequencies in patients with ovarian cancer*

Table II shows the frequencies of GSTM1, GSTT1 and CYP2D6 genotypes in controls and the 84 patients with ovarian cancer. No significant differences in genotype frequencies or frequency distributions between these groups were identified.

### *Immunohistochemical identification of p53 expression*

Sections from controls and patients with ovarian cancer were examined using the two p53 antibodies; 23 of the 63 patients (36.5%) were positive using the CM-1 antibody. In 11 of these patients, 50–75% of tumour cell nuclei stained positively for p53, while in the remaining 12, 75–100% of tumour nuclei were positive. Of these 23 patients, 18 demonstrated positivity using both CM-1 and DO-7 (Tables II and III). No cases of DO-7 positivity but CM-1 negativity were identified. All samples from controls were negative for p53 using both antibodies.

All tumour samples demonstrating p53 positivity were stage III or IV and grade 2 or 3. We did not observe a significant difference in primary response to chemotherapy with carboplatin between p53-positive and -negative cases. We also found no significant difference in histological type between p53-positive and -negative samples.

Comparison of enzyme genotypes in immunohistochemically positive and negative cases showed 20/23 CM-1-positive

**Table II** GSTM1, GSTT1 and CYP2D6 genotype frequencies in controls and patients with epithelial ovarian cancer

| | *GSTM1 A* | *GSTM1 B* | *GSTM1 A/B* | *GSTM1 null* |
|---|---|---|---|---|
| Controls ($n=312$) | 71 (22.8%) | 41 (13.1%) | 8 (2.6%) | 192 (61.5%) |
| Ovarian cancer ($n=84$) | 20 (23.8%) | 12 (14.3%) | 5 (6.0%) | 47 (55.9%) |
| p53 positive CM-1 ($n=23$) | 1 (4.3%) | 2 (8.7%) | 0 (0%) | 20 (87.0%) |
| p53 negative ($n=40$) | 14 (35.0%) | 6 (15.0%) | 4 (10.0%) | 16 (40.0%) |
| | GSTT1 null | GSTT1 positive | | |
| Controls ($n=325$) | 61 (18.8%) | 264 (81.2%) | | |
| Ovarian cancer ($n=81$) | 13 (16.0%) | 68 (84.0%) | | |
| p53 positive CM-1 ($n=22$) | 6 (27.3%) | 16 (72.7%) | | |
| p53 negative ($n=40$) | 3 (7.5%) | 37 (92.5%) | | |
| | CYP2D6 EM | CYP2D6 HET | CYP2D6 PM | |
| Controls ($n=280$) | 177 (63.2%) | 89 (31.8%) | 14 (5.0%) | |
| Ovarian cancer ($n=83$) | 49 (59.0%) | 29 (34.9%) | 5 (6.0%) | |
| p53 positive CM-1 ($n=23$) | 11 (47.8%) | 11 (47.8%) | 1 (4.3%) | |
| p53 negative ($n=40$) | 28 (70.0%) | 9 (22.5%) | 3 (7.5%) | |

Data shows genotype frequencies in controls, the total ovarian cancer case group and in the patients with ovarian cancer who demonstrated p53 immunopositivity and immunonegativity.

**Table III** GSTM1 genotypes, p53 immunoreactivity and DNA sequencing data in patients with ovarian cancer

| *GSTM1 genotype* | *GSTT1 genotype* | *CM-1 /DO-7* | *SSCP exon* | *Mutant codon* | *Nucleotide change* | *Predicted amino acid change* | *Transitn/ transvs*[a] |
|---|---|---|---|---|---|---|---|
| 1. M1 0 | T1 A | +/+ | Exon 5 | 168 | A-C/A | HIS-PRO/HIS | Transvs |
| 2. M1 0 | T1 A | +/+ | Exon 5 | 179 | C-T | HIS-TYR | Transitn |
| 3. M1 0 | T1 A | +/+ | Exon 5 | 141 | G-A/G | CYS-TYR/CYS | Transitn |
| 4. M1 0 | T1 0 | +/+ | Exon 5 | 135 | G-A | CYS-TYR | Transitn |
| 5. M1 0 | T1 0 | +/+ | Exon 6 | None | | | |
| 6. M1 A | T1 A | +/+ | Exon 7 | 248 | G-A | ARG-GLN | Transitn |
| 7. M1 0 | T1 A | +/– | Exon 7 | None | | | |
| 8. M1 0 | T1 A | +/+ | Exon 7 | None | | | |
| 9. M1 0 | T1 A | +/+ | Exon 8 | 267 | G-C | ARG-PRO | Transvs |
| | | | | 269 | A-T | SER-CYS | |
| 10. M1 0 | | +/+ | Exon 8 | 273 | G-T | ARG-LEU | Transvs |
| 11. M1 0 | T1 A | +/+ | Exon 8 | 284 | A-C | THR-PRO | Transitn |
| 12. M1 0 | T1 A | +/+ | Negative | 241 | C-T/C | SER-PHE/SER | Transitn |
| 13. M1 0 | T1 A | +/– | Negative | None | | | |
| 14. M1 0 | T1 0 | +/+ | Negative | None | | | |
| 15. M1 B | T1 A | +/+ | Negative | None | | | |
| 16. M1 B | T1 0 | +/+ | Negative | None | | | |
| 17. M1 0 | T1 A | +/+ | Negative | None | | | |
| 18. M1 0 | T1 A | +/– | Negative | None | | | |
| 19. M1 0 | T1 A | +/– | Negative | 163 | A-G/A | TYR-CYS/TYR | Transitn |
| 20. M1 0 | T1 A | +/+ | Negative | None | | | |
| 21. M1 A | T1 0 | +/+ | Negative | None | | | |
| 22. M1 0 | T1 0 | +/– | Negative | None | | | |
| 23. M1 0 | T1 A | +/+ | Negative | None | | | |

[a]Transitn, transition; transvs, transversion.

cases (87.0%) and 15/18 CM-1- and DO-7-positive cases (83.3%) were GSTM1 null (Tables II and III). Two of the other cases were GSTM1 B and one GSTM1 A. GSTM1 phenotypes in the 40 patients who demonstrated negativity for p53 are also shown in Table II. The frequency distributions of GSTM1 genotypes in the p53-positive and -negative subjects were significantly different ($\chi^2_3 = 14.15$, exact $P = 0.002$). The frequency distributions of GSTT1 genotypes in the p53-positive and -negative samples are also shown in TableII; the difference between these approached significance ($\chi^2_1 = 4.472$, exact $P = 0.057$). The number of patients with both the GSTM1 null and GSTT1 null genotypes was significantly greater in the immunopositive (4/22) than in the immunonegative group (1/40) ($\chi^2_1 = 4.708$; $P = 0.0493$). For CYP2D6, the corresponding proportions in the p53-positive cases were: CYP2D6 PM, 1/23 (CM-1) and 1/18 (CM-1 and DO-7). Frequencies of CYP2D6 genotypes in p53-positive and -negative subjects were not significantly different.

### *SSCP and DNA sequencing studies*

Table III shows the results of SSCP analysis of exons 4–9 of ovarian tumour DNA from the 23 patients who demonstrated immunohistochemical positivity for p53. A shift in the electrophoretic mobility of amplified fragments was identified in 11 patients in exons 5, 6, 7 or 8 and, in each case, this exon was sequenced. In eight of these 11 patients a mutation was found in the abnormally migrating exon, while in the remaining three patients no mutations were found. In 12 of the patients who demonstrated immunohistochemical positivity, no electrophoretic variants were identified by SSCP. Sequencing of exons 4–9 of p53 in tumour DNA from these SSCP-negative patients resulted in the detection of mutations in DNA from two patients (exons 5 and 7). No mutations were found in DNA from the remaining ten patients (Table III). Thus, in the total group of 23 patients who demonstrated immunopositivity, mutations in p53 were found in ten patients. Nine of these cases were GSTM1 null (90.0%). In the group of 13 patients in whom no mutations were identified, 11 patients were GSTM1 null (84.6%).

Table III shows the mutations and their corresponding codons found in ten of the 23 patients who demonstrated immunopositivity for p53. All mutations were missense with seven of the ten being transitions and three transversions. In four of ten tumours, both a normal and mutant allele were detected and in one case we found a mutation occurring in two separate codons (267 and 269) of p53 DNA from the same tumour.

### Discussion

We have studied the influence of polymorphism in GSTM1, GSTT1 and CYP2D6 on susceptibility to epithelial ovarian cancer in, firstly, a case–control study of genotype frequencies in patients with ovarian cancer and comparable controls, and secondly, in a study of the relationship between allelism and p53 expression. We studied protein expression using two antibodies and, in cases demonstrating immunohistochemical positivity, identified mutations by sequencing exons 4–9 of the gene in DNA from tumour material.

While the causes of ovarian cancer are unclear, the importance of ROS generated during repair of ovulation-induced damage to the ovarian surface epithelium is worthy of consideration (Goodwin *et al.*, 1993). Increased expression of p53 is likely during such oxidant stress, as DNA damage is an early consequence of exposing cells to even apparently physiological concentrations of hydrogen peroxide, presumably because of intranuclear formation of •OH. Thus, Spragg *et al* (1991) showed DNA strand breaks, 15–30 min after exposing endothelial cells to $5 \times 10^{-7}$ M hydrogen peroxide. Studies in isolated glomeruli also showed significant pyknosis, karyohexis or karyolysis after incubation with hydrogen peroxide concentrations as low as $4.7 \times 10^{-8}$ M (Clayton *et al.*, 1992). The putative role of oxidative stress in the pathogenesis of ovarian cancer suggests the importance of the GST supergene family (Strange, 1996). The isoforms encoded by GSTM1 and GSTT1 catalyse the detoxification of genotoxic chemicals, including oxidised lipid and DNA products of inflammatory stress (Strange, 1996). Homozygotes for *GSTM1*0* and *GSTT1*0* appear at increased risk of cytogenetic damage as assessed by sister chromatid exchange, and these genotypes occur with increased frequency in several cancer case groups (Smith *et al.*, 1995; Strange, 1996). We found the frequencies of GSTM1 and GSTT1 genotypes in patients with ovarian cancer were not different to those in controls, although the frequency of GSTM1 null was significantly associated with p53 immunopositivity; 20/23 of the immunopositive samples were GSTM1 null. Similarly, while not quite achieving significance, the frequency of GSTT1 null appeared greater in patients demonstrating immunopositivity (6/22). Furthermore, the frequency of the GSTM1 null/GSTT1 null haplotype was significantly increased in the immunopositive (4/22) compared with immunonegative samples (1/40). This is apparently the first report of significant interactions between these genotypes.

The p53 gene is an important target in studies assessing the consequences of allelism in carcinogen-metabolising enzymes, as over 50% of malignant tumours of different types have mutations or rearrangements of this gene (Greenblat *et al.*, 1994). Thus, inappropriate expression is found in about 50% of ovarian cancers (Eccles *et al.*, 1992*a*, *b*; Sheriden *et al.*, 1994) with studies showing a close correlation with loss of heterozygosity close to the gene; 11 of 12 tumours demonstrating immunohistochemical positivity showed LOH at 17p at the nearest informative locus to the gene. We found positivity in 36% of tumour samples examined, although as expected from published data, mutations in exons 4–9 were not identified in all of these samples (Greenblat *et al.*, 1994); we found mutations in only ten of 23 immunohistochemically positive tumours. Further, we sequenced exon–intron boundaries and found no sequence variants that would delete specific exons. We interpret these data as indicating that many of the ovarian tumours studied may overexpress wild-type p53. This view is supported by studies in a variety of cancers, in which the entire coding region of p53 has been sequenced, showing 87% of mutations are in exons 5–8 with a further 8% in exon 4 (Greenblat *et al.*, 1994). We recognise that these data, like our own, are based on sequencing within coding regions of the gene, implying they provide an underestimate of the frequency of mutations, as some occur in promoter or intronic sequences. However, this possibility appears uncommon and has not been fully evaluated in any tumour (Greenblat *et al.*, 1994).

Our data show, as expected, that patients with epithelial ovarian cancer are heterogeneous in the molecular lesions associated with carcinogenesis. In this study, some patients did not express p53 protein (although the p53 gene may carry mutations in some of these samples), others expressed persistent, mutant protein, while others appeared to overexpress detectable wild-type protein. Expression of p53, detectable by immunohistochemistry, was significantly associated with GSTM1 null. We propose this observation reflects the critical role of GSTM1 in the detoxification of the products of oxidative stress, a view supported by studies in patients with various pathologies (Strange, 1996). For example, in patients suffering systemic lupus erythematosus, GSTM1 null is associated with the photosensitivity characterised by the production of anti-Ro (but not anti-La) antibodies (Ollier *et al.*, 1996). Thus, in the ovary, chronic failure to detoxify the lipid and/or DNA products of the process of repair of the ovarian epithelium effectively may result in damage to various genes in host cells. In some patients, the p53 gene is also damaged, resulting in persistent expression of mutant protein. In other patients, the oxidative stress effects damage to various genes not including p53, resulting in overexpression of wild-type p53. The factors determining which host genes are damaged are unknown. The complexity of the ovarian cancer group is also shown by the finding that in our series, 40/63 tumours did not demonstrate p53 immunopositivity. This suggests that other tumour-suppressor genes are involved. Clearly, a better understanding of the influence of GSTM1 and other polymorphic carcinogen-metabolising enzymes on mutational events could have important implications in disease prognosis or prevention.

**Acknowledgements**
We thank Wellbeing (Royal College of Obstetrics and Gynaecologists) for a Research Training Fellowship for Mr P Sarhanis, and Professor Frank Sharp for advice and support. We also thank Mrs Nicola Buckley for skilled technical work and the North Staffordshire NHS Trust and North Staffordshire Hospital Centre Trust Fund.

### References

BELL DA, TAYLOR JA, PAULSON DF, ROBERTSON CN, MOHLER JL AND LUCIER GW. (1993). Genetic risk and carcinogen exposure: a common inherited defect of the carcinogen-metabolism gene glutathione *S*-transferase M1 (GSTM1) that increases susceptibility to bladder cancer. *J. Natl Cancer Inst.*, **85**, 1159-1164.

CLAYTON L, HILEY C, DAVIES SJ, D'SOUZA RJ, JONES P, STRANGE RC AND ABER GM. (1992). Glomerular injury induced by hydrogen peroxide: modifying influence of ACE inhibitors. *Free Rad. Res. Commun.*, **17**, 271-278.

DEAKIN M, ELDER J, HENDRICKSE C, PECKHAM D, BALDWIN D, PANTIN C, WILD N, LEOPARD P, BELL D, JONES P, DUNCAN H, BRANNIGAN K, ALLDERSEA J, FRYER A AND STRANGE R. (1996). Glutathione *S*-transferase GSTT1 phenotypes and susceptibility to cancer: studies of interactions with GSTM1 in lung, oral, gastric and colorectal cancers. *Carcinogenesis*, **17**, 881-884.

ECCLES D, BRETT L, LESSELS A, GRUBER L, LANE D, STEEL CM AND LEONARD RC. (1992*a*). Overexpression of the p53 protein and allele loss at 17p13 in ovarian carcinoma. *Br. J. Cancer*, **65**, 40-45.

ECCLES DM, RUSSELL SE, HAITES NE, ATKINSON R, BELL DW, GRUBER L, HICKEY I, KELLY K, KITCHENER H AND LEONARD R. (1992*b*). Early loss of heterozygosity on 17q in ovarian cancer. *Oncogene*, **7**, 2069-2072.

ELEXPURU-CAMIRUAGA J, BUXTON N, KANDULA V, DIAS VS, CAMPBELL D, MCINTOSH J, BROOME J, JONES P, INSKIP A, ALLDERSEA J, FRYER AA AND STRANGE RC. (1995). Susceptibility to astrocytoma and meningioma: influence of allelism of glutathione *S*-transferase, GSTT1 and GSTM1 and cytochrome P450, CYP2D6 loci. *Cancer Res.*, **55**, 4237-4239.

GOODWIN AK, TESTA JR AND HAMILTON TC. (1993). The biology of ovarian cancer development. *Cancer*, **71**, 530-536.

GREENBLAT MS, BENNETT WP, HOLLSTEIN M AND HARRIS CC. (1994). Mutations in the p53 tumor suppressor gene: clues to cancer etiology and molecular pathogenesis. *Cancer Res.*, **54**, 4855-4878.

HEAGERTY AHM, FITZGERALD D, SMITH A, BOWERS B, JONES P, FRYER A, ZHAO L, ALLDERSEA J AND STRANGE RC. (1994). Glutathione *S*-transferase GSTM1 phenotypes and protection against cutaneous malignancy. *Lancet*, **343**, 266-268.

HEAGERTY A, SMITH A, ENGLISH J, LEAR J, PERKINS W, BOWERS B, JONES P, GILFORD J, ALLDERSEA J, FRYER AA AND STRANGE RC. (1996). Susceptibility to multiple cutaneous basal cell carcinomas: significant interactions between glutathione *S*-transferase GSTM1 genotypes, skin type and male gender. *Br. J. Cancer*, **73**, 44-48.

NEBERT DW. (1994). Drug-metabolizing enzymes in ligand-modulated transcription. *Biochem. Pharmacol.*, **47**, 25-37.

OLLIER W, DAVIES E, SNOWDEN N, ALLDERSEA J, FRYER AA, JONES P AND STRANGE RC. (1996). Association of homozygosity for glutathione-S-transferase GSTM1 null alleles with the $Ro^+/La^-$ autoantibody profile in patients with systemic lupus erythematosus. *Arthritis Rheumatism*, (in press).

PERRETT CW, CLAYTON RN, PISTORELLO M, BOSCARO M, SCANARINI M, BATES AS, BUCKLEY N, JONES P, FRYER AA, GILFORD J, ALLDERSEA J AND STRANGE RC. (1995). GSTM1 and CYP2D6 genotype frequencies in patients with pituitary tumours: Effects on p53, ras and gsp. *Carcinogenesis*, **16**, 1643-1645.

RYBERG D, KURE K, LYSTAD S, SKAUG V, STANGELAND L, MERCY I, BORRESEN A-L AND HAUGAN A. (1994). p53 mutations in lung tumours: relationship to putative susceptibility markers for cancer. *Cancer Res.*, **54**, 1551-1555.

SHEPHERD JH. (1989). Revised FIGO staging for gynaecological cancer. *Br. J. Obstet. Gynaecol.*, **96**, 889-892.

SHERIDAN E, SILCOCKS P, SMITH J, HANCOCK BW AND GOYNS MH. (1994). P53 mutations in a series of epithelial ovarian cancers from the UK and its prognostic significance. *Eur. J. Cancer*, **30A**, 1701-1704.

SMITH G, STANLEY LA, SIM E, STRANGE RC AND WOLF CR. (1995). Metabolic polymorphism and cancer susceptibility. *Cancer Surveys*, **25**, 27-65.

SPRAGG RG. (1991). DNA strand break formation following exposure of bovine pulmonary artery and aortic endothelial cells to reactive oxygen products. *Am. J. Resp. Cell. Mol. Biol.*, **4**, 4-10.

STRANGE RC. (1996). Glutathione *S*-transferases and cancer susceptibility. In *Glutathoine S-transferase Structure Function and Clinical Implications*. Vermeulen NPE, Mulder GJ, Nieuwenhuyse H, Peters WHM and Van Bladeren PJ (eds) pp.239-247. Taylor and Francis: Salisbury.

WOLF CR, SMITH CA, GOUGH AC, MOSS JE, VALLIS KA, HOWARD G, CAREY FJ, MILLS K, MCKNEE W, CARMICHAEL J AND SPURR NK. (1992). Relationship between the debrisoquine hydroxylase polymorphism and cancer susceptibility. *Carcinogenesis*, **13**, 1035-1038.